# Long Noncoding RNAs in the Regulation of Oxidative Stress

**DOI:** 10.1155/2019/1318795

**Published:** 2019-02-17

**Authors:** Xiaole Wang, Chuqiao Shen, Jie Zhu, Guoming Shen, Zegeng Li, Jingcheng Dong

**Affiliations:** ^1^College of Integrated Chinese and Western Medicine, Anhui University of Chinese Medicine, No. 1, Qianjiang Road, Hefei City, Anhui Province, China; ^2^Department of Clinical Pharmacy, First Affiliated Hospital of Anhui University of Traditional Chinese Medicine, Meishan Road, Hefei City, Anhui Province, China; ^3^Institute of Integration of Traditional Chinese and Western Medicine, Anhui Academy of Chinese Medicine, No. 1, Qianjiang Road, Hefei City, Anhui Province, China; ^4^Institutes of Integrative Medicine, Fudan University, Shanghai, China; ^5^Institute of Traditional Chinese Medicine Prevention and Control on Respiratory Disease, Anhui Academy of Chinese Medicine, No. 117, Meishan Road, Hefei City, Anhui Province, China

## Abstract

Oxidative stress takes responsibility for various diseases, such as chronic obstructive pulmonary disease (COPD), Alzheimer's disease (AD), and cardiovascular disease; nevertheless, there is still a lack of specific biomarkers for the guidance of diagnosis and treatment of oxidative stress-related diseases. In recent years, growing studies have documented that oxidative stress has crucial correlations with long noncoding RNAs (lncRNAs), which have been identified as important transcriptions involving the process of oxidative stress, inflammation, etc. and been regarded as the potential specific biomarkers. In this paper, we review links between oxidative stress and lncRNAs, highlight lncRNAs that refer to oxidative stress, and conclude that lncRNAs have played a negative or positive role in the oxidation/antioxidant system, which may be helpful for the further investigation of specific biomarkers of oxidative stress-related diseases.

## 1. Introduction

Oxidative stress is described as the imbalance of the oxidation/antioxidant system caused by the accumulation of free radicals, primarily the reactive oxygen species (ROS) and the reactive nitrogen species (RNS), after the stimulation of endogenous and external environment [1]. Indisputably, oxidative stress could lead to cell death and the dysfunction of physiology, which could ascribe to DNA damage, inflammation, etc. [2, 3]. As oxidative stress has widely contributed to diverse diseases, including AD, COPD, and cardiovascular disease [4–6], specific biomarkers for the diagnosis and treatment of oxidative stress related-diseases are urgently needed. Studies have identified that oxidative stress is an important activator of some critical antioxidative pathways, whereas like Nrf2/Keap1/ARE [7] still cannot be effectively activated to eliminate free radicals to exert the protective roles. COPD, for example, has shown strengthening in oxidative stress and reduced in the expression of the Nrf2 protein as well as the antioxidant genes [8]; it is hinted that there are pivotal molecules intervened in modulating oxidative stress.

Over the past decades, advanced sequencing technologies have uncovered that approximately 2% of the genome is transcribed into messenger RNA (mRNA) and a myriad of lncRNAs are pervasively transcribed [9]. lncRNAs are characterized as transcripts with more than 200 nucleotides (nt) in length with low or no ability to encode for proteins [10]. It is appreciated that lncRNAs have relevance to DNA expression, RNA transportation, etc. through molecular interactions: RNA-protein, RNA-RNA, and so forth [11, 12]. Currently, a large portion of lncRNAs are functionally characterized; nevertheless, a limited number of annotated lncRNAs has enacted important roles in oxidative stress related-diseases like the nervous system disease, respiratory system disease, and cardiovascular system disease [13–15]. Recent reports have also revealed that lncRNAs played a negative or positive role in response to oxidative stress [16, 17], which implicates that lncRNAs as key molecules may involve in the oxidative stress field. And the low conservation and tissue-specific features of most lncRNAs suggest that lncRNAs could enact specific biomarkers for oxidative stress related-diseases [18, 19].

In the paper, we review the cis and trans pattern of lncRNAs and the oxidation/antioxidant system. More importantly, we underline the current researches of lncRNAs that involved in the oxidation/antioxidant system and synthesize their potential molecular mechanisms in modulating the oxidative stress process for further investigation.

## 2. Oxidative Stress and Nrf2/Keap1/ARE Pathway

**Figure 1:**
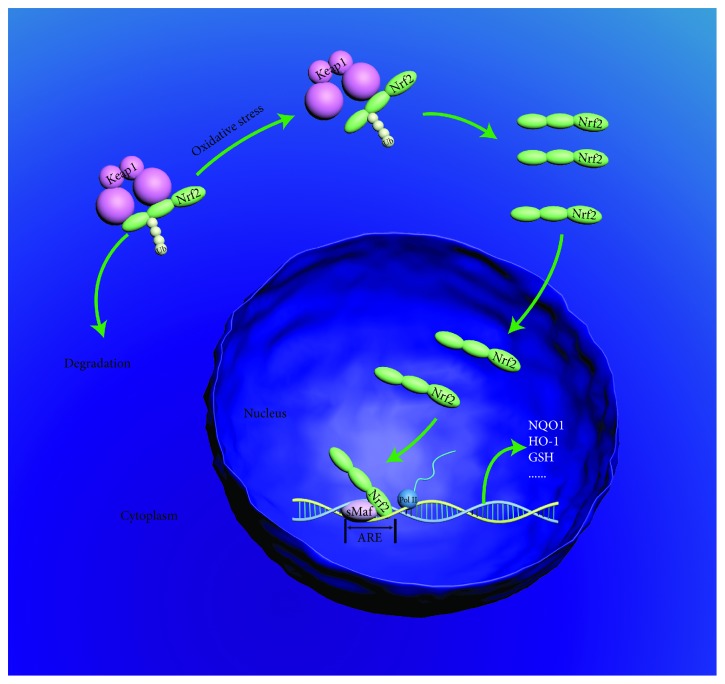
Nrf2/Keap1/ARE pathway. Under the physiological condition, Nrf2 is degraded in a Keap1 manner, whereas with the stimulation of oxidative stress, Nrf2 detaches from Keap1, enters into the nucleus, and activates the transcription of a variety of antioxidation genes.

ROS and RNS have constructed the primary proportion of free radicals (FR), which are mainly derived from the accumulation of exogenous FR such as cigarette smoking and air pollution and endogenous FR primarily released from various inflammatory cells like neutrophil and macrophage [20]. Theoretically, the performance of oxidative stress is accompanied by the activation of antioxidant pathways. For instance, the nuclear factor erythroid 2-related factor 2/kelch-like ECH-associated protein 1/antioxidant response element (Nrf2/Keap1/ARE) pathway is automatically executed in response to oxidative stress to maintain the balance of the oxidation/antioxidant system. Under physiological condition, the Nrf2, a crucial antioxidant gene activator of the Nrf2/Keap1/ARE pathway, is restrained at a low level due to the combination of Keap1. With the stimulation of oxidative stress, Nrf2 detaches from Keap1, enters and accumulates in the nucleus, and combines with ARE; afterwards, a series of antioxidative protein genes including NAD(P)H quinone dehydrogenase 1 (NQO1), heme oxygenase 1 (HO-1), and glutathione (GSH) are transcribed to maintain the balance of oxidation/antioxidant [21] (see Figure 1). But unexpectedly, a shortage of antioxidative proteins have been found in numerous diseases; recent achievements of the correlations between lncRNAs and oxidative stress may provide a novel strategy for elucidating the phenomenon.

## 3. The Pattern of lncRNA in Targeting Genes

**Figure 2:**
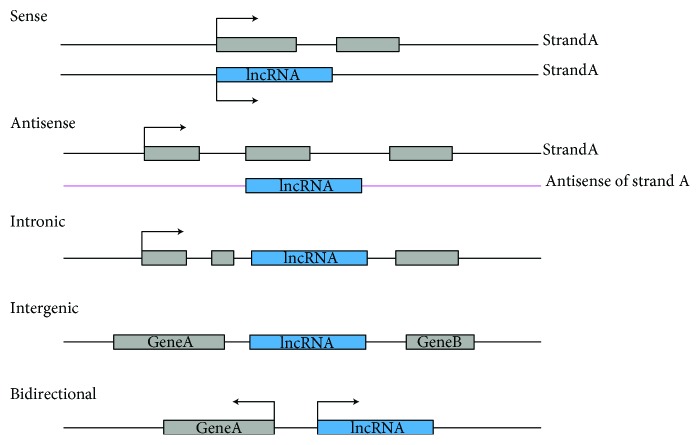
lncRNAs, according to the transcription locus relative to protein-coding genes, are classified as sense, antisense, intronic, intergenic, and bidirectional. The blue represents genes of lncRNAs; the gray represents the protein-coding genes or exons of protein-coding genes, and the arrow is the transcription direction. Sense: lncRNAs transcribe from the same strand of protein-coding genes with overlapping; antisense: lncRNAs transcribe from the antisense strand of the strand of protein-coding gene with overlapping; intron: lncRNAs entirely transcribe from the intron of protein-coding gene; intergenic: lncRNAs lie in two protein-coding genes; bidirectional: lncRNAs, in the same strand of protein-coding gene, perform the opposite transcription direction.

Currently, the criterion for the category of lncRNAs has not yet reached an agreement due to the unclear functions of numerous lncRNAs. To a consensus, lncRNAs have been classified as sense, antisense, intronic, long intergenic noncoding RNA (lincRNA), and bidirectional, according to the transcription locus [22] (see Figure 2). Besides, lncRNAs have also been categorized as signal, decoy, guide, and scaffold molecules, based on the annotated lncRNAs [23]. According to the pattern of lncRNAs regulating target genes, they are commonly identified as the cis and trans lncRNAs [24]. As the cis and trans patterns are expected to predict new and unexpected biology mechanisms of lncRNAs [25], we briefly introduce the concept of the cis pattern and trans pattern of lncRNAs with examples. The cis pattern refers to lncRNAs that regulate the adjacent protein-coding genes or chromatin status. For example, X chromosome inactivation-specific transcript (Xist), ~17 kb (in mice), ~19 kb (in human), is a lncRNA on the X chromosome, and Xist as the cis pattern lncRNAs plays a key role in X chromosome inactivation [26]. Versus the cis pattern, the trans, independent of positional relationship, refers to lncRNAs whose transcriptional locus is away from their functional locations. As an example, HOX antisense intergenic RNA (HOTAIR) is located in the human chromosome 12 with 2.2 kb in length; as a scaffold, the 5′ domain and the 3′ domain are combined with polycomb suppression complex 2 (PRC2) and LSD1/CoREST/REST complex, respectively, to reedit the chromosome state and regulate target genes [27].

## 4. lncRNAs Involving in the Oxidative Stress

Aberrant expression of lncRNAs has been observed in various diseases [28]. Heretofore, a growing number of reports have elucidated that lncRNAs have shown relevance with the oxidation/antioxidant system; most lncRNAs have been connected with the Nrf2/Keap1/ARE pathway or aimed to the miRNA to exert functions. Based on the current researches of lncRNAs in the oxidative stress field, the lncRNAs implicated with oxidative stress are listed in the table (see Table 1).

### 4.1. MALAT1

Metastasis-associated lung adenocarcinoma transcript 1 (MALAT1) is a lincRNA with 7 kb in length. It is documented that MALAT1 plays a negative or positive role in response to oxidative stress. As an activator of the antioxidant pathway, the overexpression of MALAT1 was observed in hydrogen peroxide- (H_2_O_2_-) induced human umbilical vein endothelial cells (HUVECs). Mechanically, MALAT1 lowered the Keap1 level to activate and stabilize the Nrf2 protein, thereby the antioxidant capacity was enhanced to attenuate the oxidative stress damage, lipid peroxidation, and DNA damage in H_2_O_2_-induced HUVECs [29]. MALAT1 has also been speculated as a Nrf2 regulator, which binds to Nrf2 prior to the combination of Nrf2 and ARE [30]. The regulatory interventions of MALAT1 suggest that actions on the Nrf2/Keap1/ARE pathway might be an important strategy of lncRNAs in regulating oxidative stress. In addition, the p38MAPK pathway, which has been illustrated to modulate the apoptosis and oxidative stress [31], was activated when MALAT1 was upregulated in human lens epithelial cells and binds to SP1 [32]. In addition to the above discussion, it has been observed that MALAT1 could target microRNAs (miRNAs) to alter the oxidative stress. An example is that MALAT1 showed upregulated in the brain microvascular endothelial cells under the condition of oxygen-glucose deprivation (OGD), and cells were protected from oxidative and ischemic stress damage; MALAT1 might target miR-145 to enhance the expression of VEGF-A and ANGPT2 to implement function [33]. In general, MALAT1 has implicated in oxidative stress, and various functions of MALAT1 might be due to the tissue-specific feature of lncRNAs.

### 4.2. H19

H19, 2.3 kb in length, is a highly conserved lncRNAs. Knockdown of H19 performed sensitively to H_2_O_2_, which is a common oxidative stress activator; meanwhile, six Nrf2-induced genes were reduced [34]. H_2_O_2_ could both downregulate H19 and its derived miR-675 in the cardiac progenitor cells (CPC), and the influence could be offset by the treatment of melatonin. It was further confirmed that H19-derived miR-675 targeted the 3′UTR of USP10 to downregulate p53 and p21 proteins [35]. As the MALAT1 which is mentioned above, H19 could also exert functions through miRNAs. Overexpression of H19 attenuated oxidative stress and inflammation in a diabetic mouse model, and H19 might execute the antioxidant function by targeting miR-657 to inhibit voltage-dependent anion channel 1 (VDAC1) [36]. In addition, H19, as a competing endogenous RNA, targeted IL-16 and CXCR4 to affect the invasion and migration ability of cholangiocarcinoma cells; it might enact an oxidative stress receptor for the activation of the antioxidant [37].

### 4.3. SCAL1

lncRNA SCAL1, also known as XLOC-004924 or LUCAT1, is a lincRNA located between the G protein-coupled receptor 98 (GPR98) and arrestin domain-containing 3 (ARRDC3) in human chromosome 5. SCAL1 is closely related to Nrf2, and knockdown experiments of SCAL1 or Nrf2 showed a significant increase of the toxicity of cigarette smoke (CS) in A549 cells. Meanwhile, the expression of SCAL1 was decreased, which blocked Nrf2 and further inhibited the cellular activity [38]; however, the recent studies also demonstrated that SCAL1 performed conversely functions in cancers [39, 40]. All results indicated that SCAL1 might perform a pivotal intermediate molecule in the process of Nrf2 regulating antioxidant molecules.

### 4.4. NEAT1

Nuclear-enriched abundant transcript 1 (NEAT1) is a highly conserved lincRNA. It was observed that NEAT1 could reverse the superoxide in LPS-treated rat mesangial cells [41]. NEAT1 increased the proliferation and metastasis of tumor cells and counteracted the H_2_O_2_-induced neuronal damage. The high expression of NEAT1 was also performed in enhancing the cell viability in neuro2A cells, which has showed oxidative stress and cell damage with the induction of H_2_O_2_; it suggests that NEAT1 could play as a neuroprotector in nerve injury caused by oxidative stress [42].

### 4.5. gadd7

Growth arrested DNA damage-inducible gene 7 (gadd7) is described as a contributor to DNA damage, lipotoxic stress, and nonlipid oxidative stress [43, 44]. It is the first lncRNA that presented in a feed-forward loop with oxidative stress and also enriched in ROS environments derived from lipotoxic stress. The silence of gadd7 could significantly lower ROS and delay and reduce ROS-induced endoplasmic reticulum stress [45]. Another example is that the dysregulation of gadd7 was observed in varicocele-related sperm damages [46]; as a result, gadd7 is speculated as an important participator in sperm damage caused by oxidative stress.

### 4.6. MACC1-AS1

MACC1-AS1 is transcribed from the antisense of MACC1, which is a gastric cancer metastasis-associated regulator. MACC1-AS1 has the ability to stabilize and enhance the expression of MACC1. High expression of MACC1-AS1 could promote the proliferation of gastric cancer cells, inhibit apoptosis, and regulate metabolism. Mechanistically, the AMPK/Lin28 pathway might coordinate with the process of MACC1-AS1 to enhance glycolysis and antioxidant capacity to modulate metabolic plasticity [47].

### 4.7. ODRUL

Osteosarcoma doxorubicin resistance-related upregulated lncRNA (ODRUL) has been elucidated to be induced by Nrf2 in erythroid cells treated with AgNPs. Although Nrf2 is a well-recognized antioxidant core molecule, it also shows cell damage under oxidative stress. Nrf2 promoted the transcription of ODRUL in K562 cells, thereby ODRUL interacted with PI4K*α* protein to target AKT and JNK, negatively regulated Bcl2 levels, and eventually triggered cell death [48].

### 4.8. LINC01619

LINC01619 acts as a “sponge” lincRNA of miR-27a, which has been illustrated involving endoplasmic reticulum stress and podocyte injury in diabetic nephropathy. It is speculated that LINC01619, as a competitive endogenous RNA, triggered oxidative stress and regulated miR-27a/FOXO1 to the mediation of endoplasmic reticulum stress and podocyte injury [49].

### 4.9. LINC00963

LINC00963 was initially revealed to involve in prostate cancer [50]. Blocking LINC00963 weakened the cell apoptosis, and LINC00963 might enhance the expression of FoxO3 to attenuate renal fibrosis and oxidative stress in chronic renal failure. LINC00963 is a potential marker in indicating the progression and outcome of chronic renal failure [51].

### 4.10. lnc-CD1D-2:1

lnc-CD1D-2:1, a lincRNA with two exons, has changed synchronously with ROS and performed increasingly in melanocytes irradiated by ultraviolet radiation B (UVB). Besides, lnc-CD1D-2:1, which was induced following UVB irradiation, inhibited phosphorylation of p38, and it is implied that ROS involved in UVB irradiation to produce melanin may be attributed to lnc-CD1D-2:1 [52].

### 4.11. FOXD3-AS1

FOXD3-AS1 is transcribed from the antisense of FOXD3, and it could accelerate the apoptosis of lung epithelial cells treated by oxidative stress. It has been confirmed that microRNA-150 is a protector of lung epithelial cell injury; FOXD3-AS1, as a “sponge” or the endogenous competitor of microRNA-150, blocked the protective function of microRNA-150 and enhanced the apoptosis of lung epithelial cells induced by oxidative stress [53].

### 4.12. BDNF-AS

BDNF-AS, a nature antisense lncRNA of BDNF, has been documented as a negative regulator of BDNF [54]. The content of ROS and MDA went with the expression of BDNF-AS; inversely, the antioxidant proteins like superoxide dismutase and catalase were strikingly upregulated. Induced by oxidative stress, BDNF-AS performed in decreasing cell viability and increasing cell apoptosis [55].

## 5. Conclusions

In recent years, cumulative studies have elucidated that lncRNAs, which were originally regarded as “junk” and “noise,” have widely involved in cancer, immune response, etc. [56, 57] and associated with oxidative stress [17]. Theoretically, the antioxidant system is activated in response to oxidative stress; however, crucial protective regulators like Nrf2 have shown insufficient in COPD, Alzheimer's disease, etc. [58]. Here, we highlight the lncRNAs associated with oxidative stress and present their potential mechanisms. In conclusion, lncRNAs could exert cytoprotective or damaging effects in intervening the Nrf2/Keap1/ARE antioxidant pathway, interacting with miRNA, etc. Dramatically, some lncRNAs have also performed opposite roles in different studies, which might be due to the tissue-specific feature of the lncRNAs. Our study suggests that these oxidative stress-related lncRNAs, as potential pivotal biomarkers and medicine targets, may provide a novel strategy for the diagnosis and treatment of diseases. Future studies will contribute to the precise mechanism of lncRNAs in the regulation of oxidative stress.

## Figures and Tables

**Table 1:** lncRNA involving in oxidative stress.

lncRNA	Functions	Relevant pathways	References
MALAT1	(1) Downregulating Keap1	Nrf2/Keap1/ARE	[29]
	(2) Preemptively binding with Nrf2 to inhibit the expression of Nrf2-target genes	Nrf2/Keap1/ARE	[30]
	(3) Binding with SP1	p38MAPK	[32]
	(4) Targeting miR-145 to enhance the expression of VEGF-A and ANGPT2	—	[33]
H19	(1) Antagonizing the premature senescence of CPC	—	[35]
	(2) Attenuating oxidative stress and inflammation in the diabetic mouse model	—	[36]
	(3) As a competing endogenous molecule to affect the invasion and migration ability of cholangiocarcinoma cells	—	[37]
SCAL1	Driven by Nrf2, and protecting airway epithelial cells from oxidative stress	Nrf2/Keap1/ARE	[38]
NEAT1	(1) Reversing the superoxide in LPS-treated rat mesangial cells	—	[41]
	(2) Figured as a neuroprotector in nerve injury caused by oxidative stress	—	[42]
gadd7	Induced by ROS, and low expression of gadd7 could significantly lower the ROS	—	[45]
MACC1-AS1	Promoting the proliferation of gastric cancer cells, inhibiting apoptosis, and regulating metabolism	AMPK/Lin28	[47]
ODRUL	Contributing to the toxicity in erythroid cells induced by AgNPs	Nrf2/Keap1/ARE; PI4K-AKT/JNK	[48]
LINC01619	A “sponge” of miR-27a	—	[49]
LINC00963	Attenuating renal fibrosis and oxidative stress in chronic renal	—	[51]
FOXD3-AS1	A “sponge” of microRNA-150	—	[53]
BDNF-AS	Involving in decreasing cell viability and increasing cell apoptosis induced via oxidative stress	—	[55]
